# Bioactivity of Benthic and Picoplanktonic Estuarine Cyanobacteria on Growth of Photoautotrophs: Inhibition *versus* Stimulation

**DOI:** 10.3390/md9050790

**Published:** 2011-05-10

**Authors:** Viviana R. Lopes, Vitor M. Vasconcelos

**Affiliations:** 1 CIIMAR/CIMAR, Laboratory of Ecotoxicology, Genomic and Evolution-Centre of Environmental and Marine Research, University of Porto, Rua dos Bragas 289, 4050-123 Porto, Portugal; 2 Department of Biology, Faculty of Sciences, University of Porto, Rua do Campo Alegre, 4169-007 Porto, Portugal

**Keywords:** allelopathy (negative), Atlantic estuarine environments, benthic, cyanobacteria, growth stimulation, picoplanktonic, *Phormidium* cf. *chalybeum*

## Abstract

Understanding potential biochemical interactions and effects among cyanobacteria and other organisms is one of the main keys to a better knowledge of microbial population structuring and dynamics. In this study, the effects of cyanobacteria from benthos and plankton of estuaries on other cyanobacteria and green algae growth were evaluated. To understand how the estuarine cyanobacteria might influence the dynamics of phytoplankton, experiments were carried out with the freshwater species *Microcystis aeruginosa* and *Chlorella* sp., and the marine *Synechocystis salina* and *Nannochloropsis* sp. exposed to aqueous and organic (70% methanol) crude extracts of cyanobacteria for 96 h. The most pronounced effect observed was the growth stimulation. Growth inhibition was also observed for *S. salina* and *M. aeruginosa* target-species at the highest and lowest concentrations of cyanobacterial extracts. The methanolic crude extract of *Phormidium* cf. *chalybeum* LEGE06078 was effective against *S. salina* growth in a concentration-dependent manner after 96 h-exposure. All of the cyanobacterial isolates showed some bioactivity on the target-species growth, *i.e.*, inhibitory or stimulating effects. These results indicate that the analyzed cyanobacterial isolates can potentially contribute to blooms’ proliferation of other cyanobacteria and to the abnormal growth of green algae disturbing the dynamic of estuarine phytoplankton communities. Since estuaries are transitional ecosystems, the benthic and picoplanktonic estuarine cyanobacteria can change both freshwater and marine phytoplankton succession, competition and bloom formation. Furthermore, a potential biotechnological application of these isolates as a tool to control cyanobacteria and microalgae proliferation can be feasible. This work is the first on the subject of growth responses of photoautotrophs to cyanobacteria from Atlantic estuarine environments.

## 1. Introduction

For decades, the chemistry of microorganisms and its effects on other species have been investigated. Cyanobacteria have been extensively screened and their bioactivity analyzed towards other groups. These well-known prokaryotic photoautotrophs are widespread and inhabit all kind of ecosystems. Although they can be found in terrestrial habitats, cyanobacteria are more abundant in aquatic ecosystems, where they inhabit fresh-, brackish and marine waters [1].

Cyanobacteria produce a large diversity of secondary metabolites displaying a range of biological activities and chemical structures. Some cyanobacterial secondary metabolites can exhibit toxic effects on living organisms like the common toxins microcystins [2,3]. These compounds may have ecological roles such as allelopathy being employed for biotechnological applications such as algaecides, herbicides and insecticides [4]. On the other hand, cyanobacteria have become a new source of active compounds showing interesting and exciting biological activities ranging from antibiotics, immunosuppressant, and anticancer, antiviral and anti-inflammatory to proteinase-inhibiting agents [2,3].

Most of the research conducted on biochemical impacts of cyanobacteria and their metabolites on other phytoplankton has been restricted to fresh- and marine waters. The results generally indicate that phytoplankton is negatively affected, for example by photosynthesis inhibition. Nevertheless, a handful of works shows that cyanobacteria can also stimulate phytoplankton growth [5–7]. At the present time, knowledge about bioactive effects of estuarine cyanobacteria, especially benthic forms, on phytoplankton growth is rather limited. The first work performed on brackish waters was about the Baltic Sea cyanobacteria reporting allelopathic properties of *Nodularia spumigena*, non-toxic *Aphanizomenon flos-aquae* and *Anabaena lemmermannii* [8,9]. The allelopathic effects of these species were shown by the increase of cyanobacteria, chlorophytes and chrysophytes’ growth while there was an inhibition of the cryptophytes’ growth. Nevertheless, these Baltic Sea reports only focused on the filamentous species, in particular on the toxin-producing *N. spumigena* [9,10].

The main goals of this study were to (i) assess the potential bioactivity of estuarine cyanobacteria on phytoplankton growth and (ii) evaluate how benthic and picoplanktonic forms isolated from estuarine environments may control the dynamic of phytoplankton. This was achieved by examining the effects of aqueous and lipophilic (70% methanol) crude extracts of benthic and picoplanktonic cyanobacteria on other cyanobacteria and microalgae representatives of freshwater and marine habitats. This is the first work, apart from that of the Baltic Sea waters, to evaluate crude extracts of estuarine cyanobacteria from temperate regions towards phytoplankton of different habitats.

## 2. Results and Discussion

Eighteen cyanobacterial strains, collected and isolated from Douro, Minho and Vouga Portuguese estuaries, were grown until log-phase under controlled laboratory conditions. Following, 25 mg of freeze-dried cells of each cyanobacteria culture were extracted sequentially with water and methanol: water (30:70 v/v), and reconstituted after drying. The obtained methanol and aqueous crude extracts were then screened for bioactivity towards *Chlorella* sp., *Microcystis aeruginosa*, *Nannochloropsis* sp. and *Synechocystis salina* by growth evaluation. The effects observed after 96 h-exposure and at the highest and the lowest extract concentrations were analyzed. Those were the conditions producing clearer results compared to the control. The pH was measured in the beginning and at the end of the experiment to ensure that target-species responses were not due to CO_2_ depletion. The initial pH values of target-species controls ranged from 8.17 to 9.12 and the final values between 8.94 and 9.82, without increasing more than 1 unit.

Of a total of 144 combinations, both methanol and aqueous crude extracts of estuarine cyanobacteria showed mainly stimulatory effects on the target-species growth, *Chlorella* sp., *M. aeruginosa*, *Nannochloropsis* sp. and *S. salina,* in a time-dependent manner after 96 h-exposure. However, inhibitory effects were also observed for a *Phormidium* extract against *S. salina* target-species in a time and dose-dependent manner. A wider growth inhibition was expected as reported on previous studies with pure toxins and partially purified extracts [11].

### 2.1. Growth Stimulation *versus* Inhibition

From the three main orders, Oscillatoriales, Chroococcales and Nostocales, to which the isolates were assigned, the isolates of Oscillatoriales order were the most important inducers of growth stimulation. Specifically, the aqueous extracts of the two isolates of *Leptolyngbya* sp. LEGE06069 and 07091 induced a 100-fold increase of *M. aeruginosa* growth (Table 1).

Even so, few cases of inhibition growth were also observed at the lowest and highest values of extract concentration (see Table 1). But, in both cases the effects observed occur in a concentration–response independent manner.

The *S. salina* target-species was inhibited to the greatest extent by the Oscillatoriales extracts (see Table 1). The aqueous and methanolic extracts of *Leptolyngbya, Microcoleus, Phormidium* isolates (in particular, LEGE06069, 06070, 06078, 07075, 07076, 07080, 07085, 07091 and 07092) induced mostly negative effects towards the *Chlorella* sp., *M. aeruginosa* and *S. salina* at the lowest extract concentration. The same effects have also occurred at the highest concentration of methanolic extracts of *Phormidium* and *Leptolyngbya* genera, in particular, isolates LEGE06078, 07085 and 07091. Furthermore, the target-species *Nannochloropsis* growth was also inhibited by: (i) the highest concentration of aqueous extract of *Leptolyngbya* af. *bijjugata* LEGE07075 and of methanolic extracts of both *Phormidium* isolates LEGE06072 and 06078 and (ii) by aqueous extract of *Leptolyngbya* sp. LEGE07085 at the lowest concentration.

The isolates from the other orders, Nostocales and Chroococcales (Tables 2 and 3, respectively), had also stimulated growth but only up to 10-fold. All these stimulatory effects were observed to the highest concentration of extract used (25.00 mg. mL^−1^). But, in several cases, stimulatory effects were also observed with the lowest extract concentration as described above.

The growth stimulation induced by several cyanobacteria of the three orders can be explained as a nutritional effect of compounds present in the cell extracts as well as an adaptation approach of target organisms to the potential allelochemicals [12]. Furthermore, the ability of the target strains to metabolize the allelochemicals-like compounds present can be also an explanation for the stimulatory growth effects [13]. A low concentration of potential allelopathic substances can as well justify the predominance of stimulatory effects (at the lowest and highest extract concentrations) since it may not be high enough to inhibit the growth of target-species, as previously shown to other kinds of interactions [14]. Besides, we cannot exclude the potential effects of phytohormones such as auxins, gibberellins, cytokinins, which are also synthesized and liberated by cyanobacteria. Several works reported the stimulatory effects of phytohormones on plants [15]. In addition, Ravikumar [16] studied the effects of phytohormones, such as indole-3 acetic acid, on cyanobacteria growth showing the same growth promoting effects to those previously reported on plants.

Concerning the isolates of Nostocales, data point out that *Chlorella* sp. and *Nannochloropsis* sp. are the less negatively influenced target-species (see Table 2).

For the lowest concentration of both aqueous and methanolic extracts of *Nodularia* sp. LEGE06071 and *Nostoc* sp. 06077 isolates, growth inhibition was the main effect observed towards *S. salina* and *M. aeruginosa*. Additionally, both concentrations of LEGE06077 aqueous extract inhibited the *Nannochloropsis* growth, while only the highest concentration (25.00 mg.mL^−1^) inhibited *S. salina* growth. These findings differ from the recent results with *Gloeotrichia echinulata* (Nostocales), which was shown to increase the growth rates of phytoplankton species (microalgae and cyanobacteria) up to 620% [17].

The estuarine extracts of Chroococcales inhibited mostly *S. salina* growth (Table 3). Interestingly, the inhibition of *S. salina* target-species growth was induced by isolates of the same species (LEGE06079, 06083 and 07073) independently of the extract or the concentration value (maximum or minimum) tested. These findings might suggest a strategy to avoid intra-group competition.

Those previous extracts also produced inhibitory effects on the target-species growth, *M. aeruginosa*, although only observed at the lowest concentration. Furthermore, inhibitory effects on the target-species *Chlorella* and *Nannochloropsis* growth were also observed to the lowest concentration of aqueous and methanolic extracts of *Synechocystis* and *Synechococcus* sp. (LEGE 07073 and 07074 respectively).

Overall, it seems that cyanobacterial target-species are the most sensitive to the extracts of their congeners, which suggest that these biochemical interactions can be group-specific.

The fact that same cyanobacterial isolates can produce either stimulatory or inhibitory effects suggests that: (i) different compounds, sharing the same target or not may coexist, leading to synergistic effects, or (ii) the same compound might differently affect the different target-species [14,18]. Synergy is a well-known phenomenon in the context of complex mixtures such as cell extracts [19]. Chemicals found to inhibit the growth of a species at a certain concentration/dose may stimulate the growth of the same or other species at a lower concentration [20,21]. The different sensitivity of the target species to the concentration threshold used may also enlighten the contrasting effects [14].

Since we used cyanobacterial extracts, a mixture of compounds, these findings of stimulatory effects on phytoplankton could be due to the fact that: (i) many phytoplankton species can use cyanobacterial secondary metabolites for their own metabolism; (ii) some cyanobacterial compounds have antibacterial or antifungal activities which might benefit the phytoplankton; (iii) cyanobacteria can also produce extracellular growth promoting compounds such as phytohormones [16,22,23]. In fact, the different pattern of response observed with several targets might suggest that the involved compounds may be specific [24] but further studies with more target species have to be done for clarification.

Besides, the role of heterotrophic bacteria present in the non-axenic cultures cannot be discarded. These bacteria are known to degrade cyanobacterial chemicals and, in addition, to release either their own antibiotics or stimulatory compounds [18]. Even so, the presence of heterotrophic bacteria mimics the field conditions being closer to natural conditions.

### 2.2. Potential Allelopathic Effects

**Figure 1.**
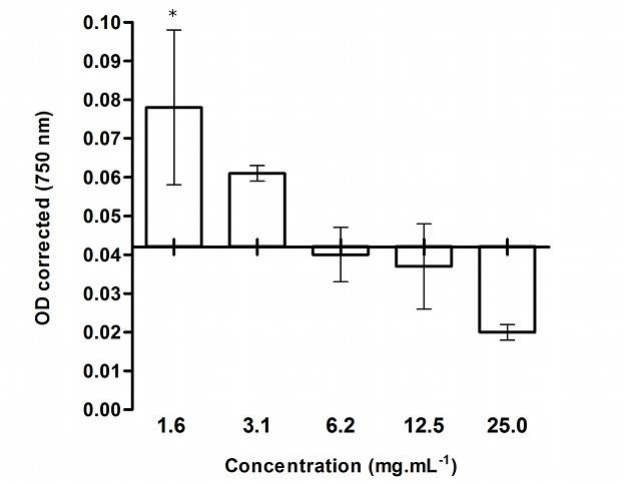
Inhibition of *Synechocystis salina* growth by the methanolic extract *Phormidium* cf. *chalybeum* LEGE06078 at 96 h exposure. Statistical significance is marked with an asterisk (*p* < 0.05). The control value is represented by x-axis (OD ∼ 0.04).

Among these extracts, only the crude methanolic extract of *Phormidium* cf. *chalybeum* (LEGE06078) showed inhibitory effects towards *S. salina* growth in a concentration-dependent manner after 96 h-exposure (Figure 1).

This kind of response, a biphasic dose-response, is characterized by low dose stimulation or beneficial effect and a high dose inhibitory effect, which is a fundamental feature of hormesis. The term hormesis has been widely applied in toxicology and can be defined as a process in which exposure to a low dose of a chemical agent or environmental factor that is damaging at higher doses induces an adaptive beneficial effect on the cell or organism [25].

The inhibitory effects observed are in accordance with previous studies involving growth effects of *Phormidium* species against other cyanobacteria. In fact, Volk [26] described negative (allelopathic) interactions of *Phormidium foveolarum* against filamentous cyanobacteria. Furthermore, Valdor and Aboal [11] had also reported the cyanobacterial growth inhibition after exposure to methanolic extracts of *Phormidium* sp. Besides, the fact of the cyanobacterial isolate LEGE06078 being a filamentous form, it is also in agreement with previous studies showing inhibitory effects (algicidal) induced by filamentous cyanobacteria [18].

The observed effects are clear hints of potential allelopathy of estuarine cyanobacteria against their counterparts. Further studies are needed to clarify if the potential allelochemicals are being released to the medium even if these substances may be available during cell lyses.

We suggest that the potential allelochemicals-like compounds involved might have a lipophilic nature rather than hydrophilic since the extract tested was prepared with 70% of methanol. Despite the fact that lipophilic compounds on aquatic systems are more specific when the compounds act by direct contact although this is not the case (interaction of between benthic donors and planktonic target-species), they cannot be excluded. Besides, the low solubility of the compounds can be circumvented by small particles binding or micelles formation [27,28].

This study shows that the cyanobacteria from estuaries, mainly benthic forms, might influence and interact either by stimulating or inhibiting growth of planktonic microalga (including cyanobacteria). Likewise, Mohamed [29] showed that a benthic cyanobacterium stimulated the growth of a natural planktonic cyanobacterial community from irrigation canals, even if the collection site is different from estuaries.

## 3. Experimental Section

### 3.1. Cyanobacterial Isolates

The cyanobacteria tested in this study were collected and isolated from three estuaries of the north and center of Portugal, namely Douro, Minho and Vouga river estuaries, for which salinity ranged from 0.5 to 35.0. The source of the 18 isolates studied and their morphological identification are given in Table 4.

In brief, the cyanobacteria cultures were isolated by standard microbiological methods and maintained in Z8 [30] with or without addition of NaCl (10 to 35 g.L^−1^), when required, or BG11 medium [31] at 25 °C under a light/dark cycle of 14/10 h and 20.8–27.4 × 10^−6^ μmol.m^2^.s^−1^ photon irradiance.

### 3.2. Extracts Preparation

The methanolic (70%) and aqueous extracts were prepared as described previously [32]. Briefly, each cyanobacterial culture in log-phase growth was collected and freeze-dried. Then, the freeze-dried cells were extracted with each respective solvent applying an ultrasonic probe Vibra cell50 (Sonics & Materials Inc. Danbury, USA) at 4 °C during 3 × 20 seconds, centrifuged at 9 300 *g* during 10 minutes and evaporated. The obtained residue of each extract were evaporated, dissolved in medium with DMSO (P.A. Sigma-Aldrich, USA) and filtered through 0.45 and 0.22 μm filters at a final concentration of 25.00 mg of biomass per mL of solvent. The final DMSO concentration on the medium was 0.1% (v/v) *i.e.*, 100 μL DMSO per 100 mL medium. Herein, the extract weight corresponds to the freeze-dried cells weight before extraction and not to the weight of extract itself. A two-fold dilution series of the cyanobacterial extracts (1.56, 3.12, 6.25, 12.50, 25.00 mg mL^−1^) was analyzed in triplicate.

### 3.3. Microalgal Growth Assay

#### 3.3.1. Target Strains

The freshwater *Microcystis aeruginosa* LEGE91094 (non-microcystin producer/class Cyanophyceae) and *Chlorella* sp. LEGE Z-001 (Chlorophyceae), and the marine *Synechocystis salina* LEGE06099 (Cyanophyceae) and *Nannochloropsis* sp. LEGE Z-004 (Eustigmatophyceae) were obtained from Laboratory of Ecotoxicology, Genomic and Evolution (LEGE) culture collection. The species were maintained in Z8 medium [30] or Z8 medium containing 20 mg.mL^−1^ of NaCl (Z_8_^20^) depending if they were from fresh or marine waters, respectively, at 25°C under a light/dark cycle of 14/10 h and 20.8–27.4 × 10^−6^ μmol.m^2^.s^−1^ photon irradiance. The organisms were grown as monoclonal and non-axenic cultures. The LEGE collection is maintained in LEGE laboratory, CIIMAR, University of Porto.

#### 3.3.2. Assessment of Microalgal Growth

A preliminary curve-growth of each target-species was performed assessing growth by optical density measurement of the culture at 750 nm with a PowerWave™ Microplate Spectrophotometer (BioTek, USA) as well as by cell counting. It was experimentally verified that a linear relationship exists between cell density (cell.mL^−1^) and optical density:

Cell density *Chlorella* (cell.mL^−1^) = 3E+07 * A750 nm + 3.28E+05 (r^2^ = 0.999)
Cell density *Nannochloropsis* (cell.mL^−1^) = 4E+07 * A750 nm – 6.48E+04 (r^2^ = 0.997)
Cell density *Microcystis* (cell.mL^−1^) = 4E+08 * A750 nm + 6.54E+05 (r^2^ = 0.999)
Cell density *Synechocystis* (cell.mL^−1^) = 5E+08 * A750 nm + 4.00E+06 (r^2^ = 0.998)

#### 3.3.3. Bioassay

The microplate-based assay was performed as described previously by Gantar *et al.* [18] with slight modifications. Briefly, a suspension of 100 μL of each log-phase growing microalgae-stock cultures (*ca*. 5 × 10^5^ cell.mL^−1^) was added in each well containing 100 μL of the extract ressuspended in specific medium (plus DMSO) previously filtered. The microplates were observed daily under optical microscope to verify if no bacterial growth was present.

Growth during the 96 h exposure period was measured daily, included after the inoculation of target species, by optical density (OD) at 750 nm. Target microalgae cultures with no tested extracts and with medium plus DMSO (not exceeding 100 μL DMSO per 100 mL of medium) were used as control and solvent control, respectively. The pH in the control cultures was evaluated in the beginning and at the end of the test with a 3510 pH Meter (Jenway, UK) at room temperature. Due to the short-term nature of our experiments, no measurements of medium nutrient concentrations were done. All treatments were evaluated in three replicates.

#### 3.3.4. Statistical Analysis

The optical density (OD) of each treatment was corrected with the OD measured after the inoculation of the target species to reduce background. The growth fold induction/inhibition at 96 h was calculated as followed: (OD treatment corrected/OD control). Regarding the inhibition growth plot, a statistical significance of the different means observed was determined by one-way analysis of variance (ANOVA) followed by the Dunnett’s Multiple Comparison Test. The tests were performed using SPSS 14.0 after the homoscedasticity of the variance was checked. A significant level of *p* < 0.05 was accepted.

## 4. Conclusions

Concluding, this study is the first of its kind to focus mainly on the effects of benthic cyanobacteria from Atlantic estuarine environments on microorganisms belonging to different compartments of the ecosystem. Considering that estuaries are transitional ecosystems between the ocean and freshwater biome, the benthic and picoplanktonic estuarine cyanobacteria can alter phytoplankton succession, competition and bloom formation.

The impacts of estuarine cyanobacteria on surrounding biota can have two hypothetical scenarios: (i) the estuarine cyanobacteria can produced compounds that potentially benefit phytoplankton growth (specifically other cyanobacteria and microalgae) disturbing the structure and dynamic of some estuarine phytoplankton communities, and consequently enhance eutrophication; (ii) they can be a natural control factor of phytoplankton proliferation. Therefore, the potential bioactive compounds present in the extracts of estuarine cyanobacteria may be used as biotechnological tools to limit phytoplankton proliferation. Further studies on the identification and characterization of potential allelochemicals produced by estuarine cyanobacteria are needed.

## Figures and Tables

**Table 1.** Growth responses of the target-species, *Nannochloropsis* sp. (N), *Chlorella* sp. (C), *Synechocystis salina* (S) and *Microcystis* sp. (M) exposed to aqueous (WE) and methanolic (ME) estuarine extracts of Oscillatoriales at 96 h. Fold induction based on optical densities (relative to the control) is shown to the highest (25.00) and lowest (1.56 mg.mL^−1^) concentration value of extracts.

**Isolate LEGE/Species name**	**Extract**	**Conc. (mg.mL ^−1^)**	**Growth fold induction ^a^**			
			**N ^b^**	**C**	**S**	**M**
06069 *Leptolyngbya* sp.1	ME ^c^	H ^d^	1.4	5.9	3.0	3.9
		L	1.0	**0.4 ^e^**	1.3	**0.9**

	WE	H	2.2	6.2	6.9	103.5 ^f^
		L	1.3	1.6	**0.6**	19.0

06070 *Leptolyngbya* sp*.2*	ME	H	20.4	1.6	2.9	6.4
		L	10.2	1.0	**0.9**	2.1

	WE	H	10.2	1.3	2.4	1.9
		L	10.9	1.1	1.4	1.6

06072 *Phormidium* cf. *animale*	ME	H	**0.3**	1.2	**0.6**	7.1
		L	2.1	1.1	1.1	1.7
	WE	H	4.7	1.3	1.6	7.8
		L	3.7	1.0	1.2	1.6

**06078** *Phormidium* cf. *chalybeum*	ME	H	**0.7**	2.5	**0.4**	9.5
		L	4.2	1.2	1.9	2.7

	WE	H	10.0	2.0	1.6	5.5
		L	3.3	1.3	**0.9**	1.3

07080 *Leptolyngbya* sp.1	ME	H	15.7	2.5	3.3	5.2
		L	7.4	1.2	**0.4**	1.0

	WE	H	24.2	2.5	2.4	16.3
		L	6.7	1.0	**0.3**	3.9

07075	ME	H	7.9	1.7	2.3	4.5

*Leptolyngbya* sp.2		L	5.0	1.1	1.3	1.5

	WE	H	**0.5**	**0.4**	**0.5**	3.3

		L	4.0	1.1	1.3	2.1

07076	ME	H	8.5	1.4	1.9	5.8

*Microcoleus vaginatus*		L	5.1	1.1	1.3	2.9

	WE	H	41.7	1.9	2.7	8.6

		L	9.5	**0.6**	**0.5**	4.4

07084 *Leptolyngbya* sp.1	ME	H	6.3	2.0	4.6	21.8
		L	1.5	1.8	1.2	2.9

	WE	H	1.7	1.5	2.8	6.8
		L	1.4	1.4	1.2	2.7

07085 *Leptolyngbya* aff. *bijugata*	ME	H	1.3	1.7	**0.8**	3.5
		L	1.9	1.2	1.4	1.4

	WE	H	7.8	1.2	**0.5**	3.6
		L	**0.7**	1.1	1.4	1.2

07091 *Leptolyngbya* sp.1	ME	H	3.0	**0.9**	10.3	74.3
		L	2.1	1.3	**0.9**	20.3

	WE	H	8.3	1.4	22.2	123.0*
		L	2.6	1.2	**0.9**	34.3

07092 *Microcoleus chthonoplastes*	ME	H	2.2	5.9	3.0	4.5
		L	1.3	**0.4**	1.3	1.6

	WE	H	20.4	6.2	6.9	7.1
		L	10.2	1.6	**0.6**	1.7

a Fold induction was calculated as: Fold induction = (OD treatment corrected/OD control). Controls here used were the target-species without extract.

b N—*Nannochloropsis* sp.; C—*Chlorella* sp.; S—*Synechocystis salina*; M—*Microcystis aeruginosa*.

c ME—methanolic extract; WE—aqueous extract.

d H—25.00 mg of biomass per mL and L—1.56 mg of biomass per mL.

e The inhibition cases are marked in boldface.

f. The highest values of growth fold induction (growth stimulation).

**Table 2.** Growth responses of the target-species *Nannochloropsis* sp. (N), *Chlorella* sp. (C), *Synechocystis salina* (S) and *Microcystis* sp. (M) exposed to aqueous (WE) and methanolic (ME) estuarine extracts of Nostocales at 96 h. Fold induction based on OD (relative to the control) is shown to the highest (25.00) and lowest (1.56 mg.mL^−1^) concentration value of extracts. Footnotes explanation can be seen with Table 1.

**Isolate LEGE/Species name**	**Extract**	**Conc. (mg.mL^−1^)**	**Growth fold induction**			
			**N**	**C**	**S**	**M**
06071 *Nodularia* sp.	ME	H	9.8*	1.6	5.7	3.8
		L	6.9*	1.4	**0.6**	**0.3**

	WE	H	3.8	1.8	1.9	2.8
		L	3.1	1.7	**0.1**	**0.6**

06077 *Nostoc* sp.	ME	H	3.5	**0.8**	**0.1**	**0.5**
		L	2.3	1.1	**0.0**	**0.3**

	WE	H	**0.6**	1.0	**0.4**	2.7
		L	**0.4**	1.0	**0.2**	**0.5**

**Table 3.** Growth responses of the target-species *Nannochloropsis* sp. (N), *Chlorella* sp. (C), *Synechocystis salina* (S) and *Microcystis* sp. (M) exposed to aqueous (WE) and methanolic (ME) estuarine extracts of Chroococcales at 96 h. Fold induction based on OD (relative to the control) is shown to the highest (25.00) and lowest (1.56 mg.mL^−1^) extract concentrations. Footnotes explanation can be seen with Table 1.

**Isolate LEGE/Species name**	**Extract**	**Conc. (mg.mL^−1^)**	**Growth fold induction**			
			N	**C**	**S**	**M**
06068 *Cyanobium* sp.	ME	H	5.4	2.6	3.6	6.4
		L	1.5	1.1	1.0	1.5

	WE	H	4.9	3.4	3.8	8.3*
		L	1.5	1.4	**0.8**	**0.7**

06079 *Synechocystis salina*	ME	H	5.0	2.6	**0.6**	3.4
		L	3.5	1.1	**0.0**	**0.0**

	WE	H	2.1	3.4	**0.4**	2.9
		L	2.1	1.4	**0.1**	**0.3**
	
06083 *Synechocystis* cf. *salina*	ME	H	5.0	1.9	3.2	7.8*
		L	3.5	1.1	**0.3**	**0.2**

	WE	H	2.1	1.7	**0.5**	1.7
		L	2.1	**0.8**	**0.5**	**0.2**

07073 *Synechocystis* cf. *salina*	ME	H	2.6	1.2	**0.6**	3.4
		L	1.6	**0.9**	**0.0**	**0.0**

	WE	H	7.9*	2.2	**0.4**	2.9
		L	**0.4**	1.0	**0.0**	**0.3**

07074 *Synechococcus* sp.	ME	H	6.2	1.8	4.3	1.1
		L	**0.3**	1.2	1.7	3.9

	WE	H	**0.5**	2.4	**0.7**	2.6
		L	2.2	1.3	1.6	1.5

**Table 4.** Origin and identification of the cyanobacterial isolates from Atlantic estuaries used in this study.

**Order No. LEGE**	***Taxa (genus/species)***	**Habitat**	**Coordinates source (Lat./Lon.)**	
Chroococcales				
06068	*Cyanobium* sp.	benthos	N 41° 8′ 50. 77″	W 8°39′ 12. 89″
06079	*Synechocystis salina*	benthos	N 41° 8′ 12. 20″	W 8°39′ 54. 65″
06083	*Synechocystis* cf. *salina*	picoplankton	N 41° 8′ 48. 17″	W 8°39′ 3 8. 79″
07073	*Synechocystis* cf. *salina*	picoplankton	N 40° 40′ 16. 42″	W 8°43′ 24. 36″
07074	*Synechococcus* sp.	benthos	N 41° 8′ 48. 17″	W 8°39′ 3 8. 79″
Oscillatoriales				
07075	*Leptolyngbya* sp.2	benthos	N 41° 8′ 50. 45″	W 8°3 8′ 2. 13″
07080	*Leptolyngbya* sp.1	benthos	N 41° 52′ 2. 50″	W 8°51′35. 90″
07084	*Leptolyngbya* sp.1	benthos	N 41° 52′ 16. 76″	W 8°50′ 39. 66″
07085	*Leptolyngbya* aff. *bijugata*	benthos	N 41° 8′ 50. 77″	W 8°39′ 12. 89″
07091	*Leptolyngbya* sp.1	benthos	N 40° 40′ 16. 42″	W 8°43′ 24. 36″
06069	*Leptolyngbya* sp.1	benthos	N 41° 8′ 50. 45″	W 8°3 8′ 2. 13″
06070	*Leptolyngbya* sp*.2*	benthos	N 41° 8′ 50. 45″	W 8°3 8′ 2. 13″
07076	*Microcoleus vaginatus*	benthos	N 41° 54′5. 00″	W 8°4 8′ 51. 88″
07092	*Microcoleus chthonoplastes*	benthos	N 40° 40′ 16. 42″	W 8°43′ 24. 36″
06072	*Phormidium* cf. *animale*	benthos	N 40° 40′ 16. 42″	W 8°43′ 24. 36″
06078	*Phormidium* cf. *chalybeum*	benthos	N 41° 8′ 12. 20″	W 8°39′ 54. 65″
Nostocales				
06071	*Nodularia* sp.	benthos	N 40° 38′ 32. 87″	W 8°39′ 47. 85″
06077	*Nostoc* sp.	picoplankton	N 41° 52′ 40. 13″	W 8°50′ 6. 33″
